# Content of Health Economics Analysis Plans (HEAPs) for Trial-Based Economic Evaluations: Expert Delphi Consensus Survey

**DOI:** 10.1016/j.jval.2020.10.002

**Published:** 2021-04

**Authors:** Joanna C. Thorn, Charlotte F. Davies, Sara T. Brookes, Sian M. Noble, Melina Dritsaki, Ewan Gray, Dyfrig A. Hughes, Borislava Mihaylova, Stavros Petrou, Colin Ridyard, Tracey Sach, Edward C.F. Wilson, Sarah Wordsworth, William Hollingworth

**Affiliations:** 1 Population Health Sciences, Bristol Medical School, University of Bristol, Bristol, UK; 2 Oxford Clinical Trials Research Unit, Nuffield Department of Orthopaedics, Rheumatology, and Musculoskeletal Sciences, University of Oxford, Oxford, UK; 3 Edinburgh Clinical Trials Unit, University of Edinburgh, Edinburgh; 4 Centre for Health Economics and Medicines Evaluation, Bangor University, Bangor, UK; 5 Health Economics Research Centre, Nuffield Department of Population Health, University of Oxford, Oxford, UK; 6 Institute of Population Health Sciences, Barts and The London School of Medicine and Dentistry, Queen Mary University of London, London, UK; 7 Oxford National Institute for Health Research Biomedical Research Centre, The Joint Research Office, Oxford, UK; 8 Nuffield Department of Primary Care Health Sciences, University of Oxford, Oxford, UK & Warwick Clinical Trials Unit, Warwick Medical School, University of Warwick, Coventry, UK; 9 Health Economics Group, Norwich Medical School, University of East Anglia, Norwich, UK

**Keywords:** analysis plans, bias, economic evaluation

## Abstract

**Objectives:**

Health economics analysis plans (HEAPs) currently lack consistency, with uncertainty surrounding appropriate content. We aimed to develop a list of essential items that should be included in HEAPs for economic evaluations conducted alongside randomized trials.

**Methods:**

A list of potential items for inclusion was developed by examining existing HEAPs. An electronic Delphi survey was conducted among professional health economists. Respondents were asked to rate potential items from 1 (least important) to 9 (most important), suggest additional items, and comment on proposed items (round 1). A second survey (round 2) was emailed to participants, including the participant’s own scores from round 1 along with summary results from the whole panel; participants were asked to rerate each item. Consensus criteria for inclusion in the final list were predefined as >70% of participants rating an item 7-9 and <15% rating it 1-3 after round 2. A final item selection meeting was held to scrutinize the results and adjudicate on items lacking consensus.

**Results:**

62 participants completed round 1 of the survey. The initial list included 72 potential items; all 72 were carried forward to round 2, and no new items were added. 48 round 1 respondents (77.4%) completed round 2 and reached consensus on 53 items. At the final meeting, the expert panel (n = 9) agreed that 58 items should be included in the essential list, moved 9 items to an optional list, and dropped 5 items.

**Conclusions:**

Via expert consensus opinion, this study identified 58 items that are considered essential in a HEAP.

## Highlights

• Health economics analysis plans (HEAPs) are becoming best practice for economic evaluations conducted alongside randomized controlled trials; however, they are currently implemented inconsistently.
• This study used Delphi consensus techniques to identify a list of items that should be included in a HEAP.
• A standardized HEAP will contribute to the transparency and reproducibility of economic evaluations and to the minimization of bias.

## Introduction

The use of statistical analysis plans (SAPs) is an accepted means of reducing bias in randomized controlled trials (RCTs) by minimizing selective reporting of results and undeclared post hoc analyses. A SAP is defined as "a document that contains a more technical and detailed elaboration of the principal features of the analysis described in the protocol, and includes detailed procedures for executing the statistical analysis of the primary and secondary variables and other data".^1^

Economic evaluations are frequently conducted alongside RCTs, providing evidence of the value for money that a health intervention offers to inform funding allocation decisions. SAPs are used routinely in RCTs by trial statisticians. However, in contrast to SAPs, health economics analysis plans (HEAPs) are not universally implemented by health economists, are not mandated in SPIRIT (Standard Protocol Items: Recommendations for Interventional Trials^2^) or other such guidelines,^1^ and lack standardization in their content. A recent survey of current practice found that only around 30% of 28 responding clinical trials units in the UK always use some form of HEAP, and there was little consistency in the approach taken by different units.^3^

In October 2015, a workshop funded by the Medical Research Council was held in Bristol, UK, to provide a forum in which issues around current practice and opinions on the appropriate use of HEAPs could be discussed. The 50 participants (who were mainly UK-based health economists) contributed to various group discussions including a debate on the appropriate content of HEAPs, which highlighted the range of opinions and the need to achieve consensus. Feedback from the workshop suggested that most participants considered that developing a HEAP had some merits in trial-based economic evaluations. Furthermore, there was a recognized need for robust guidance on the content of HEAPs.^4^

Guidance for the content of SAPs has recently been developed and identifies 61 items that are considered essential for inclusion.^5^ It also provides a consensus opinion that some additional trial-based analyses, including statistical procedures for the health economics component of the study, should not be included within the SAP. Prespecified analysis plans have been used in other areas of economics^6^; however, guidance on preparing HEAPs for economic evaluations conducted alongside RCTs and their appropriate content is currently sparse.^7^ HEAPs are rarely published; the few examples are typically standalone appendices^8^ or part of a SAP,^9^ and their content varies. This article aims to specify the preferred content of HEAPs for use with RCTs using a consensus approach to identify the appropriate items. To help prevent bias in trial-based economic evaluations, it is important that good-quality HEAPs are developed and adopted more widely.

## Methods

Ethics approval for the study was granted by the Faculty of Health Sciences Research Ethics Committee of the University of Bristol (application reference number: 54903). Analyses were carried out using Microsoft Excel 2016.

Consensus on the essential items for inclusion in a HEAP was sought using Delphi methodology,^10^ which has been widely employed in recent years in a number of different healthcare contexts.^11^ In a Delphi study, a panel of experts in the field is asked to express an opinion on the question being studied. These results are summarized at a group level and participants are asked to reconsider their original opinions in light of group feedback. The process is repeated iteratively until a prespecified consensus point is reached. Participants generally remain anonymous and independent of one another, ensuring that more assertive group voices do not dominate the decision-making process.

### Identification of “Long List” of Items and Development of the Delphi Survey

Current examples of HEAPs were solicited from health economists registered on the HEALTHECON-ALL mailing list (approximately 2500 international members with diverse backgrounds in terms of their level of experience, work setting, and methodological expertise).^12^ An initial list of items for consideration was identified from these sources and supplemented with information from UK-based clinical trial unit HEAP standard operating procedures (SOPs) supplied by request to the project team. The project team met to discuss the “long list,” and items were examined for significant overlap. Similar items were combined; for example, “Scope of HEAP,” “General principles,” and “Purpose of HEAP” were combined as “Purpose of HEAP.” Items were split if they covered 2 or more distinct topics. Each item was briefly summarized in a statement (eg, the item “Valuation of resource-use data” was summarized as “For each resource measured, describe how the unit cost will be derived”), and an example of how the item might appear in a HEAP was prepared based on excerpts from the HEAP examples. This list was then used to form the basis of a 2-round electronic Delphi survey, which was developed using REDCap data capture software^13^ and hosted at the University of Bristol. Items were grouped into thematic sections within the survey, each of which was presented to participants on a separate page. Only fully completed surveys were analyzed. Both rounds of the survey were tested and piloted by the project team, and improvements in wording to aid clarity were made after piloting.

### Participants and Recruitment

Potential Delphi participants were recruited using the HEALTHECON-ALL mailing list through a generic invitation to participate in the survey by following a web link. Email invitations (n = 90) were also sent directly to health economists, including attendees of the 2015 HEAPs workshop in Bristol, and to appropriate unit leads in the UK and internationally. Prospective participants were asked to have had some experience with HEAPs. Completion of the first round of the HEAPs Delphi survey was considered to represent informed consent to participate in the project. Participants’ professional details were requested at the start of the survey and included number of years’ experience in health economics, number of HEAPs worked on, country of work, and type of economic analysis mainly undertaken by the participant.

### Delphi Survey

#### Round 1

Participants were asked to rate each item on a Likert scale from 1 to 9, where 1 represented an item that is nonessential and 9 represented an item that must be included in a HEAP. Free-text boxes at the end of each section allowed participants to suggest additional items and provided an opportunity to add any further comments or feedback. A reminder email was sent to the HEALTHECON-ALL mailing list 2 days before the 2-week deadline in the original email. Results from round 1 were analyzed and used to develop the list of items to be included in a second round. The number and percentage of people rating an item as of high importance (defined as 7 to 9) and as of low importance (defined as 1 to 3) were calculated, along with the mean (standard deviation) and median score for each item. Criteria for dropping items prior to round 2 were predefined based on previous published work.^5^ Items were dropped if >50% of participants rated an item 3 or lower and <15% rated it 7 or higher. A new item would be added to the second round if it was suggested by more than 10% of respondents.

#### Round 2

**Figure 1:**
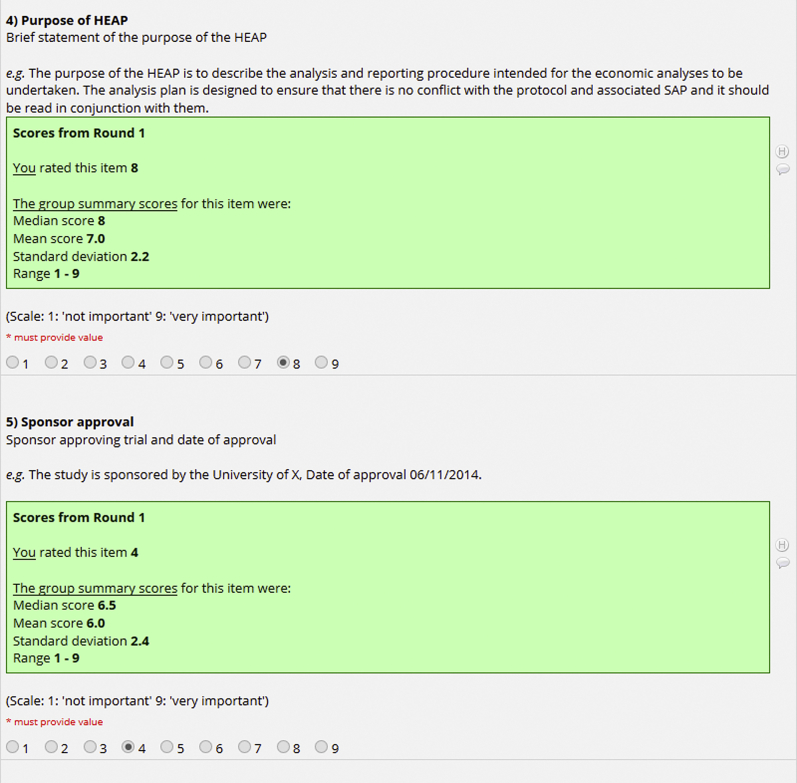
Extract from round 2 of Delphi survey.

All participants who had completed round 1 of the survey were emailed a web link to complete a personalized version of round 2 (Fig. 1) within a specified 2-week time period for response. Summary statistics of the round 1 results were presented (mean, standard deviation, median, range), alongside a reminder of the participant’s own response in round 1. Minor changes to wording from round 1, made on the basis of the feedback provided, were highlighted in the round 2 survey. Participants were asked to rerate each item taking into consideration the feedback from round 1. The option to provide qualitative feedback was provided at the end of the second-round survey, and participants were asked to confirm whether they would be available and willing to attend a final item selection meeting. Where required, a reminder email was sent 2 days before the 2-week survey submission deadline, specifying the closing date. If participants did not respond after the reminder email, they were contacted by telephone, using publicly available work contact information.

Following round 2, agreement was assessed using the RAND/UCLA approach, which takes into account not only the score itself, but also the degree of dispersion among responses.^14^ The proportion of respondents rating items high and low were calculated, and the predefined consensus criteria (Table 1) were applied. For round 2, items were determined as having achieved “consensus in” if 70% or more participants scored the item as 7-9 and less than 15% of participants scored the item as 1-3. The criteria were the same as those used to determine the content of SAPs for randomized controlled trials^5^ and in other contexts (eg, ^15^). However, as these criteria are essentially an arbitrary choice, the RAND/UCLA appropriateness method^14^ (which combines the RAND/UCLA agreement measure with the median score) was used to inform discussion in the final item selection meeting. Medians of 7-9 alongside agreement suggested inclusion, medians of 1-3 alongside agreement suggested exclusion, and medians of 4-6 with or without agreement represented an unclear outcome.

**Table 1:** Predefined consensus classification criteria for round 1 (R1) and round 2 (R2) of the Delphi survey.

Consensus classification	Description	Definition
Consensus in	Consensus that component should be included in the HEAP	50% (R1) or 70% (R2) or more participants scoring as 7 to 9 AND <15% participants scoring as 1 to 3
Consensus out	Consensus that component should not be included in the HEAP	50% (R1) or 70% (R2) or more participants scoring as 1 to 3 AND <15% participants scoring as 7 to 9
No consensus	Uncertainty about importance of component	Anything else

### Final Item Selection Meeting

After round 2 of the Delphi survey, a final item selection meeting was held in Bristol (March 19, 2018) to discuss the results of the Delphi survey and to reach an inclusion decision on items lacking consensus. The meeting was attended by the following expert panel: the HEAP project team (comprising 8 health economists and one Delphi coordinator), 2 Delphi participants, and 2 clinical trial unit representatives. A Delphi expert was available for consultation by telephone if required. Items where consensus had been achieved were discussed briefly for group confirmation and agreement. The expert panel members were asked to vote on items that had not reached consensus in the Delphi survey. After discussion of the reasons for or against inclusion, panel members voted using TurningPoint software to allow for anonymous responses.^16^ The panel was asked to vote for one of the following possible outcomes: (1) to keep an item in the essential HEAP list; (2) to place the item on an optional list; or (3) to take the item out of the HEAP completely. Votes were cast by the 9 health economists (except the meeting chair) present. Items were selected (or otherwise) on the basis of a simple majority; where there was a tie, further discussion took place before a repeat vote was taken.

## Results

### Initial “Long List”

106 potential items for inclusion in the Delphi survey were extracted from a total of 18 HEAPs and 4 SOPs. Following deduplication and merging of similar items, an initial long list of 72 items was created, and organized into 8 sections: Administrative information, Trial introduction and background, Economic approach/overview, Economic data collection and management, Economic data analysis, Modeling and value of information analyses, Reporting/publishing, and References and appendices.

### Delphi Survey

**Figure 2:**
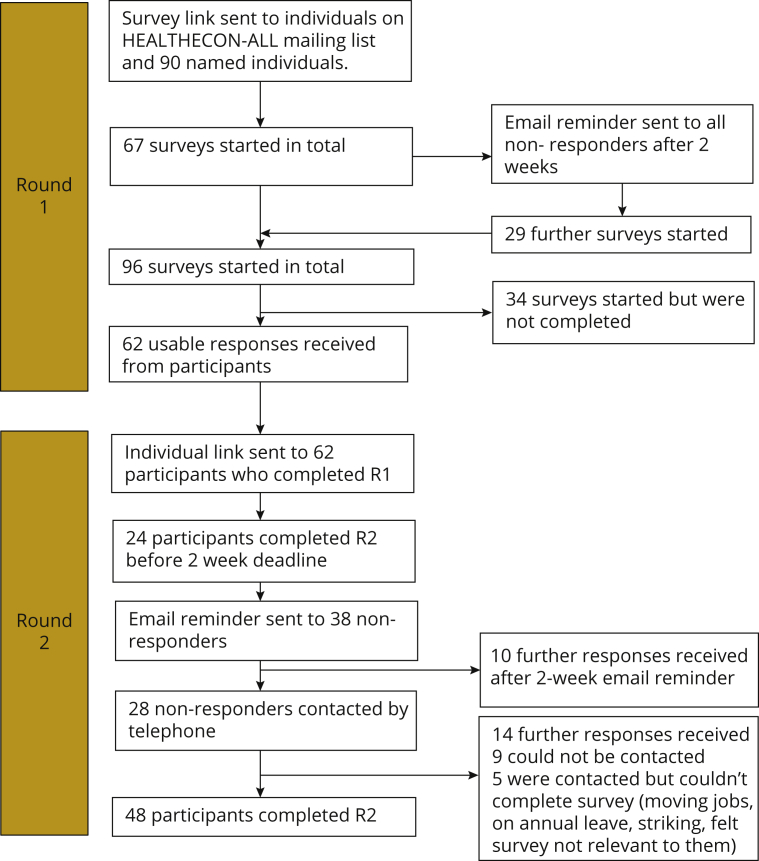
Delphi survey participant numbers. (R1 = round 1, R2 = round 2).

The numbers of participants responding to each round of the survey are shown in Fig. 2. Piloting of the round 1 survey indicated that it was relatively straightforward to understand, taking around 20 minutes to complete. Sixty-two participants provided complete responses to round 1. Most participants carried out their health economics work in the UK (77%), other European countries (16%), and Australasia (5%); other participant characteristics are shown in Table 2. Comments from participants included suggestions for new items and improvements to wording or examples, justifications and explanations for particular ratings, and some concern over duplication with material in other trial documentation. Comments on the modeling section drew attention to the difficulty of prespecifying model analyses that may be dependent on trial results.

**Table 2:** Characteristics of Delphi panel participants.

	Round 1 (n = 62)	Round 2 (n = 48)
	Number (%)	Number (%)
Country of health economics work		
UK	48 (77.4)	38 (79.1)
Other Europe	10 (16.1)	7 (14.6)
Austra/Asia	3 (4.8)	2 (4.2)
Other	1 (1.6)	1 (2.1)
Professional background		
Health economist	60 (96.8)	47 (97.9)
Other	2 (3.2)	1 (2.1)
Number of HEAPs experienced		
0	4 (6.5)	4 (8.3)
≤5	24 (38.7)	17 (35.4)
>5	32 (51.6)	25 (52.1)
No answer	2 (3.2)	2 (4.2)
Main work setting		
Academia	53 (85.4)	40 (83.3)
Industry	4 (6.5)	3 (6.3)
Other	4 (6.5)	4 (8.3)
No answer	1 (1.6)	1 (2.1)
Main analysis type		
Within trial analysis	25 (40.3)	21 (43.7)
Model based on a trial	8 (12.9)	6 (12.5)
Both	29 (46.8)	21 (43.7)
Years in health economics		
<5	11 (17.7)	7 (14.6)
5-10	14 (22.6)	13 (27.1)
11-20	26 (41.9)	21 (43.7)
>20	11 (17.7)	7 (14.6)

The mean (standard deviation) item score in round 1 was 7.3 (2.1), with individual item means ranging between 5.1 (2.3) and 8.8 (0.5). Applying the predefined consensus criteria for round 1 (>50% rating an item 7-9, <15% rating it 1-3) resulted in 56 items remaining in the HEAP. Sixteen items did not reach consensus, and no items met the criteria for being dropped (>50% rating the item 1-3 and <15% rating it 7-9); full round 1 results are given in Table 3. None of the new items proposed by participants in the comments sections met the inclusion requirement of being suggested by >10% of participants. Therefore, because no items were voted out in round 1, all 72 items were carried forward to round 2, allowing participants to rate all items in context. A small number of changes (n = 7) were made to the wording of items.

**Table 3:** Results from the Delphi survey.

Item	Round 1 Median score	Round 1 Item IN/OUT or NO CONSENSUS	Round 2 Median score	Round 2 Number (%) rated 7 to 9	Round 2 Number (%) rated 1 to 3	Round 2 Item IN/OUT or NO CONSENSUS	Item status after final voting
Title	8	IN	8	39 (81.3)	3 (6.3)	IN	IN
Trial registration number	8	IN	8	42 (87.5)	1 (2.1)	IN	IN
Source of funding	8	IN	8	40 (83.3)	2 (4.2)	IN	IN
Purpose of health economics analysis plan (HEAP)	8	IN	8	37 (77.1)	2 (4.2)	IN	IN
Sponsor approval	6.5	NO CON	6	14 (29.2)	5 (10.4)	NO CON	OUT
Trial protocol version	7	IN	7	37 (77.1)	1 (2.1)	IN	IN
Trial statistical analysis plan (SAP) version	7	IN	7	34 (70.8)	1 (2.1)	IN	IN
Trial HEAP version	8	IN	8	42 (87.5)	1 (2.1)	IN	IN
HEAP revisions	7	IN	7	35 (72.9)	2 (4.2)	IN	IN
Table of contents	6	NO CON	5	11 (22.9)	9 (18.8)	NO CON	OPTIONAL LIST
Abbreviations/glossary of terms/definitions	6.5	NO CON	6	18 (37.5)	5 (10.4)	NO CON	OPTIONAL LIST
Roles and responsibilities	7	IN	7	33 (68.8)	0 (0)	NO CON	IN
Signature(s) of person(s) writing HEAP (and date)	6	NO CON	6	16 (33.3)	9 (18.8)	NO CON	IN
Signature of senior health economist (HE) who is guarantor of the economic evaluation (and date)	6	NO CON	6	18 (37.5)	6 (12.5)	NO CON	IN
Signature of the chief investigator for the trial	6	NO CON	6	15 (31.3)	9 (18.8)	NO CON	IN
Trial background and rationale	7	IN	7	35 (72.9)	2 (4.2)	IN	IN
Aim(s) of the trial	8	IN	8	38 (79.2)	1 (2.1)	IN	IN
Objectives and/or research hypotheses of the trial	7	IN	8	37 (77.1)	2 (4.2)	IN	IN
Trial population	7	IN	7	41 (85.4)	2 (4.2)	IN	IN
Intervention and comparator(s)	9	IN	9	47 (97.9)	1 (2.1)	IN	IN
Trial design	8	IN	8	45 (93.8)	1 (2.1)	IN	IN
Trial start and end dates	7	IN	7	33 (68.8)	3 (6.3)	NO CON	IN
Aim(s) of economic evaluation	9	IN	9	48 (100)	0 (0)	IN	IN
Objectives(s)/hypotheses of economic evaluation	9	IN	9	47 (97.9)	0 (0)	IN	IN
Overview of economic analysis	9	IN	9	48 (100)	0 (0)	IN	IN
Jurisdiction	7	IN	7	38 (79.2)	2 (4.2)	IN	IN
Perspective(s)	9	IN	9	47 (97.9)	0 (0)	IN	IN
Time horizon	9	IN	9	48 (100)	0 (0)	IN	IN
Monitoring collection of health economic data	7	NO CON	7	34 (70.8)	4 (8.3)	IN	OPTIONAL LIST
Database management	7	NO CON	7	25 (52.1)	5 (10.4)	NO CON	OPTIONAL LIST
Data entry	7	NO CON	6	23 (47.9)	4 (8.3)	NO CON	OPTIONAL LIST
Data cleaning for analysis	7	IN	7	33 (68.8)	5 (10.4)	NO CON	IN
Data archiving	6.5	NO CON	6	17 (35.4)	4 (8.3)	NO CON	OPTIONAL LIST
Statistical software used for HE analysis	7	IN	7	35 (72.9)	3 (6.3)	IN	IN
Identification of resources	9	IN	9	47 (97.9)	0 (0)	IN	IN
Measurement of resource use data	9	IN	9	48 (100)	0 (0)	IN	IN
Valuation of resource use data	9	IN	9	45 (93.8)	0 (0)	IN	IN
Identification of outcome(s)	9	IN	9	48 (100)	0 (0)	IN	IN
Measurement of outcome(s)	9	IN	9	48 (100)	0 (0)	IN	IN
Valuation of outcome(s)	9	IN	9	47 (97.9)	0 (0)	IN	IN
Analysis population	9	IN	9	45 (93.8)	0 (0)	IN	IN
Timing of analyses	8	IN	8	40 (83.3)	1 (2.1)	IN	IN
Discount rates for costs and benefits	9	IN	9	43 (89.6)	1 (2.1)	IN	IN
Cost-effectiveness threshold(s)	8.5	IN	8	39 (81.3)	4 (8.3)	IN	IN
Statistical decision rule(s)	8	IN	8	39 (81.3)	3 (6.3)	IN	IN
Analysis of resource use	9	IN	9	43 (89.6)	0 (0)	IN	IN
Analysis of costs	9	IN	9	46 (95.8)	0 (0)	IN	IN
Analysis of outcomes	9	IN	9	47 (97.9)	0 (0)	IN	IN
Missing data	9	IN	9	48 (100)	0 (0)	IN	IN
Analysis of cost-effectiveness	9	IN	9	47 (97.9)	0 (0)	IN	IN
Sampling uncertainty	9	IN	9	47 (97.9)	0 (0)	IN	IN
Subgroup analysis/Analysis of heterogeneity	9	IN	9	45 (93.8)	0 (0)	IN	IN
Sensitivity analyses	9	IN	9	47 (97.9)	0 (0)	IN	IN
Post hoc analyses	8	IN	8	38 (79.2)	2 (4.2)	IN	OUT
Extrapolation or decision analytic modeling	9	IN	9	46 (95.8)	0 (0)	IN	IN
Model type	9	IN	9	44 (91.7)	1 (2.1)	IN	IN
Model structure	8.5	IN	8	44 (91.7)	1 (2.1)	IN	IN
Treatment effect beyond the end of the trial	8.5	IN	8	42 (87.5)	2 (4.2)	IN	IN
Other key assumptions	8	IN	8	41 (85.4)	1 (2.1)	IN	IN
Methods for identifying and estimating parameters	8	IN	8	43 (89.6)	1 (2.1)	IN	IN
Model uncertainty	9	IN	9	45 (93.8)	1 (2.1)	IN	IN
Model validation	8	IN	8	42 (87.5)	1 (2.1)	IN	IN
Subgroup analyses/Heterogeneity	8	IN	8	41 (85.4)	2 (4.2)	IN	IN
Value of information analysis	6.5	NO CON	6	20 (41.7)	6 (12.5)	NO CON	OPTIONAL LIST
Responsibility for health economic results and reporting	7	NO CON	6.5	24 (50)	4 (8.3)	NO CON	OUT
Reporting standards	7	IN	7	35 (72.9)	2 (4.2)	IN	IN
Reporting deviations from the HEAP	8	IN	8	40 (83.3)	2 (4.2)	IN	IN
References to trial and statistical master file	6	NO CON	6	13 (27.1)	9 (18.8)	NO CON	OUT
References to other trial documents	6.5	NO CON	6	19 (39.6)	5 (10.4)	NO CON	OPTIONAL LIST
Appendices: Resource use data collected	7	IN	7	33 (68.8)	4 (8.3)	NO CON	IN
Appendices: Reporting checklists	6	NO CON	6	13 (27.1)	12 (25)	NO CON	OUT
Appendices: Illustrations	5	NO CON	5	3 (6.3)	13 (27.1)	NO CON	OPTIONAL LIST

Round 2 of the survey was completed by 48/62 participants (77.4%). Nonresponders to round 2 (n = 14) rated items in round 1 at a mean of 7.6 compared to 7.3 for responders (*P* = .34). Reasons given over the telephone from nonresponders for not completing round 2 included changing jobs, on annual leave, on strike (UK academics were in dispute with employers over changes to the academic pension scheme at the time of the survey), or they felt the survey was not relevant to them. Nine participants could not be contacted by telephone. RAND/UCLA approach agreement criteria^14^ were met for all of the items, and standard deviations were lower for all items in round 2 than in round 1. The mean (standard deviation) item score in round 2 was 7.4 (1.9). Comments in round 2 focused on explaining some of the scores given, and they reiterated an intention to avoid overlap with other trial documentation. The issue of divergent results and conclusions (ie, an analysis that does not find a clinical outcome difference between intervention and comparator but finds that the intervention is likely to be cost-effective) was also raised.

At the end of round 2, 53 items met the consensus conditions for appearing in the final HEAP template and 19 items did not reach consensus. No items met the consensus criteria for being dropped from the HEAP.

### Final Item Selection Meeting

Discussions at the final item selection meeting resulted in 5 items being voted out of the HEAP (Table 3). Of these, 4 had not reached consensus in the Delphi survey; votes were 8 in favor of dropping the item and 1 in favor of putting it on the optional list in each case. The remaining item (“post hoc analyses”) had reached the consensus criteria for inclusion in the Delphi survey. However, following discussion of the merits of its inclusion, the group felt that post hoc changes (ie, those occurring after any analysis has begun, or study intervention allocation has been unblinded) should be documented in the trial outputs rather than in the HEAP itself, and that a priori changes (ie, before first analysis) to the HEAP were already covered in the administrative section. The item was therefore voted out by a majority: 6 votes for exclusion and only 3 votes for inclusion.

For 7 items that had not reached consensus in the Delphi survey, voting indicated that they should be included in the final HEAP. For 1 of these items (“Data validation and cleaning”), the inclusion vote was dependent on a change in wording and a move to a different section. Nine items were not voted essential but were considered suitable for an optional list; of these, 8 had not reached consensus in the Delphi survey, and the ninth only just achieved consensus.

A total of 58 items were therefore designated for inclusion in the HEAP essential list. The final outcomes for each item are given in Table 3. The current HEAP template headings, descriptions, and example texts are given in the Supplemental Materials found at https://doi.org/10.1016/j.jval.2020.10.002; updated versions with refinements to the examples elicited through practical application of the template will be deposited with the University of Bristol research repository.^17^

## Discussion

In this study, we used Delphi methodology to derive a consensus opinion on a minimum set of items that should be included in a HEAP for an economic evaluation conducted alongside an RCT. The 58 selected items form a coherent set of information covering administrative details, trial particulars, details of the economics study methods (including data collection, data management and analysis, and modeling approaches), and reporting aspects. A further 9 items were deemed important to consider including in a HEAP, although not essential. Good agreement on the relative importance of individual items was found among participants in the Delphi survey.

The study benefited from a panel of health economists from a variety of backgrounds, many of whom had substantial experience of working with HEAPs. Although drawn from an international pool, the participants were predominantly from the UK and working in academia. The use of the international HEALTHECON-ALL mailing list explicitly targeted broader participation; however, the preliminary workshop in 2015 had already indicated that HEAPs were less widely used alongside RCTs outside the UK, and even less so outside Europe. Although the UK-centric nature of the participants may have an impact on the generalizability of the results, we believe that the template will have applicability beyond the UK. The participation of the project team in the final-item selection meeting is not expected to have led to bias in the selected items given the prospective specification of the process and full description of results at each step. Consensus criteria were defined in advance of the survey in order to adhere to established Delphi methods. The sample size of 48 individuals completing both rounds is reasonable, given that even small sample sizes for Delphi studies can give reliable outcomes.^18^ Two rounds of the Delphi survey were adequate, as agreement based on the RAND/UCLA criteria was achieved for all items following the second round. Owing to the timing of the survey, which spanned the UK Christmas holiday season, the 2 rounds were held two and a half months apart; however, participants in round 2 were reminded of their own scores from the first round, attrition was relatively low (over 75% of respondents completed both rounds), and there was no evidence from comments in the second round that the timing was an issue.

The inclusion of modeling items led to considerable discussion, both among the project team and in the comments provided within the Delphi survey. Respondents pointed out that it is not uncommon for model structures to change during a project as evidence external to the trial evolves; prespecifying methods could be unhelpfully limiting for model analyses given that modeling is carried out for the purpose of decision analysis rather than trial reporting and must be responsive to the information needs of decision makers. However, all the modeling items (except value of information items) were rated highly in the Delphi survey with consensus that they should be included in a HEAP (although it should be noted that approximately 40% of the participants worked mainly on within-trial economic analyses). As described by one survey participant, this section should be treated as a model conceptualization rather than a prescriptive outline of the entire model, particularly given that the evidence required for a model may not be available at the time of writing the HEAP; the examples given alongside the items were constructed to demonstrate this.

Concerns over the credibility of clinical trial and other scientific results have led to calls for adopting measures designed to improve conduct and reporting of studies.^19^ The suite of documentation that is now associated with RCTs (including the protocol, SAP, data-sharing requirements, and reporting of results) aims to improve the transparency and reproducibility of clinical research. Preparation of the HEAP should be carried out in the context of other trial documentation and in consultation with statisticians to ensure consistency of approaches and terminology with the SAP. In this study, we aimed to identify a comprehensive list of items that could be used in a standalone HEAP. Many of the items are administrative or relate to the trial itself rather than the economic evaluation specifically. There is inevitably some overlap with the SAP and other documentation, and, in practice, some items could be cross-referenced.

Attendees at the 2015 HEAPs workshop felt that HEAPs need to be implemented with some flexibility. Where changes are necessary, the final study report should acknowledge and justify any deviations from the HEAP, such as changes in best practice methods. Flexibility may also be required for novel trial designs; although the list identified here was not developed with any particular trial design in mind, it may be necessary to add additional items (eg, for adaptive or factorial trials). It is also important to note that writing a HEAP requires time that must be accounted for in resourcing projects. Finally, the guidance produced for SAPs states that “SAPs should be made publicly available.”^5^ In line with this advice, we suggest the same should apply to HEAPs, and that HEAPs (if not published) should be deposited in accessible repositories.

While the key purpose of a HEAP is to increase transparency and reduce the potential for bias, preparation of a HEAP confers additional benefits. For example, it can increase the efficiency of the analysis phase, highlight potential issues at an early stage, and foster good communication between health economists and the wider trial team. In the event of staff turnover, a comprehensive HEAP can protect the trial team against knowledge loss. It may be necessary to revise the list of essential HEAP items in the future to reflect conceptual, methodological, and practical advances or changes in the field.

## Conclusions

The aim of a HEAP is to prevent bias in the results of economic evaluations arising from selective reporting or analyses being cherry-picked once the data have been examined, and to enable reproducibility. However, it is also important that the documents are not overly bureaucratic and burdensome for researchers. This study generated 58 core items that were considered essential for inclusion within a HEAP via expert consensus opinion. The list captures all the most important items but is considered a manageable size; these essential items form a template HEAP that will provide guidance for economic evaluations in RCTs. HEAPs should now be implemented more widely and consistently by the health economics community.
