# Supplementary material for: Content of Health Economics Analysis Plans (HEAPs) for Trial-Based Economic Evaluations: Expert Delphi Consensus Survey
Source: Value Health. 2021 Apr;24(4):539–47. doi: 10.1016/j.jval.2020.10.002 (PMC8024512; doi:10.1016/j.jval.2020.10.002)
Supplement: Supplemental Material [file mmc1.docx]

**Supplementary material 1. Health Economics Analysis Plan (HEAP) template v1.0**

The full list of essential and optional items is given below, with expanded item descriptions and practical examples of how the item might appear in a HEAP. Please note that the examples are drawn from a number of different studies.^†^

**Essential items**

|  |  | **Description** | **Example** |
| --- | --- | --- | --- |
| **Section 1: Administrative information** | | | |
| 1.1 | Title | Title that matches protocol and which includes the phrase ‘Health Economics Analysis Plan' | Health economics analysis plan for the [trial name]: a multi-centre randomised controlled trial to assess whether X improves the outcome of Y |
| 1.2 | Trial registration number | Trial registration number and name of registry that uniquely identifies the clinical trial on a publicly accessible registry (and other relevant trial study numbers) | ISRCTNXXXXXXXX (ISRCTN registry) |
| 1.3 | Source of funding | Name of funders for trial and economic evaluation and funder(s)’ reference number(s) | National Institute for Health Research, Health Technology Assessment; Reference number – XX/XX/XX |
| 1.4 | Purpose of HEAP | Brief statement of the purpose of the HEAP | The purpose of this HEAP is to describe the analysis and reporting procedure intended for the economic analyses to be undertaken. The analysis plan is designed to ensure that there is no conflict with the protocol and associated statistical analysis plan and it should be read in conjunction with them. |
| 1.5 | Trial protocol version | Trial protocol version number associated with this HEAP | This document has been written based on information contained in the trial protocol version 4, dated 14 October 2019 |
| 1.6 | Trial Statistical Analysis Plan (SAP) version | SAP version number associated with this HEAP | SAP Version: 1.0, Date: 4 August 2019 |
| 1.7 | Trial HEAP version | Sequential number and date of this version | HEAP Version: 1.0, Date: 4 August 2019 |
| 1.8 | HEAP revisions | Date, justification for revision and summary of changes to the HEAP. Specify the individual making any revisions/changes to the HEAP. | *The HEAP revision history can be presented in tabular format with the following column headings:*  Updated HEAP Version No; Protocol version; Section number changed; Description of, and reason for, change; Individual making the change (e.g. Dr [name] made the revision before any comparative analyses had been conducted); Date changed.  Each row subsequently added to the table will indicate each HEAP revision change. |
| 1.9 | Roles and responsibilities | Names, affiliations and roles of individuals who have significantly contributed to the HEAP | This HEAP was prepared by Dr [name] (junior health economist) and approved by, Prof/Dr [name] (senior health economist). The trial health economist(s) [name (s)] are responsible for conducting and reporting the economic evaluation in accordance with the HEAP. |
| 1.10a | Signature(s) of person(s) writing HEAP | Signature(s) of the person(s) writing the HEAP (and date) |  |
| 1.10b | Signature of senior health economist | Signature of senior health economist who is guarantor of the economic evaluation (and date) |  |
| 1.10c | Signature of Chief Investigator | Signature of the Chief Investigator for the trial (and date) |  |
| **Section 2: Trial introduction & background** | | | |
| 2.1 | Trial background and rationale | Synopsis of trial background and rationale including a brief description of research question and brief justification for undertaking the trial | Testing the effectiveness of new school-based educational interventions for children and young people with depression is a priority. The [trial name] is a pragmatic, parallel design, two arm, multicentre randomised controlled trial to determine whether a group-based CBT course is more effective than curriculum as usual for people who have had a diagnosis of depression in the past 24 months. |
| 2.2 | Aim(s) of the trial | Clearly and briefly state the main aim(s) of the trial | Briefly, the [trial name] RCT aims to inform primary care prescribing practice by investigating the severity and duration of depressive symptoms that are associated with a clinically significant response to sertraline compared to placebo, in people presenting to primary care with depression. |
| 2.3 | Objectives and/or research hypotheses of the trial | Describe specific trial objectives (primary and secondary) or trial hypotheses | Primary objective: to assess whether there is a clinically important difference in the impact of fatigue 26 weeks after baseline (i.e. starting the intervention) between patients participating in a cognitive-behavioural course delivered by the clinical team compared with patients receiving usual care. Secondary objectives: to compare differences between groups for secondary outcomes of fatigue severity, sleep, pain, disability, health service use, quality of life and cost-effectiveness for the NHS, patients and society. |
| 2.4 | Trial population | Describe the trial inclusion and exclusion criteria | Inclusion criteria: GP patients with a diagnosis of severe mental illness. Raised total cholesterol above 5 or raised total cholesterol above 4 and one or more of the following risk factors: Current smoker, BMI>30kg/m^2^, diagnosis of diabetes and aged 30-75 years old.  Exclusion criteria: Under acute psychiatric care. Life expectancy of less than 6 months. Pregnant at baseline. Unable to give informed consent. |
| 2.5 | Intervention(s) and comparator(s) | Describe the intervention(s) and comparator(s) | Intervention: Patients in the GP practices allocated to the intervention will be offered an enhanced cardiovascular disease risk reducing service over 6 months. The intervention will include one or more of the following elements: lifestyle advice, education on diet and exercise, behavioural techniques to encourage patients to make healthy lifestyle changes, signposting to smoking cessation or physical activity programmes.  Comparator: Treatment as usual. |
| 2.6 | Trial design | Briefly describe the trial design including type of trial such as cluster, crossover, etc. Can also include details of power calculation, sample size (including any separate calculations for economic endpoints), randomisation and blinding. | This is a non-blinded cluster RCT with GP practices being randomised to the intervention or treatment as usual on a 1:1 basis. Randomisation of GP practices will be carried out by an independent statistician. A total of 500 patients from 75 GP practices will be recruited into the trial. |
| 2.7 | Trial start and end dates | Trial recruitment start and end dates and the follow-up period | Recruitment started in October 2017 and is due to finish in October 2020. The follow-up period will run for 12 months until October 2021. |
| **Section 3: Economic approach/overview** | | | |
| 3.1 | Aim(s) of economic evaluation | Describe the aim(s) of the economic evaluation | The aim of the economic evaluation is to address the question “What is the cost-effectiveness of continuous subcutaneous insulin injections (CSII) compared with multiple daily injections (MDI) in patients with type 1 diabetes receiving a structured education programme?”. |
| 3.2 | Objective(s) of economic evaluation | Describe the objectives (primary and secondary) of the economic evaluation | The primary objective of the health economic evaluation is to estimate the long-term cost-effectiveness of continuous subcutaneous insulin injections (CSII) versus multiple daily injections (MDI) for patients receiving a structured education programme over a life time horizon using economic modelling techniques. A secondary objective is to calculate the short-term cost-effectiveness of CSII versus MDI in the first 2 years after patients receive the education programme in a within-trial economic evaluation. |
| 3.3 | Overview of economic analysis | Briefly outline and justify the type of economic evaluation to be undertaken, identifying the primary economic analysis and outlining the analysis plan and the methods that will be used | The within-trial economic analysis will be performed using individual patient level data from the [trial name]. The analytical approaches will take the form of cost-effectiveness and cost-utility analysis. Based on trial evidence, incremental cost-effectiveness (and cost-utility) ratios will be calculated by taking a ratio of the difference in the mean costs and mean effects (or utility measure). |
| 3.4 | Jurisdiction(s) | Specify the jurisdiction(s) in which the analysis will be conducted including details of the country(s) and health system(s) | The trial is conducted in the UK which has a national health service (NHS), providing publicly funded healthcare, primarily free of charge at the point of use. |
| 3.5 | Perspective(s) | State the perspective(s) from which the economic analysis is being conducted, such as societal perspective and/or healthcare payer perspective | The primary economic analysis will be from the NHS and personal social services (PSS) perspective. A secondary analysis will include the perspective of patients and carers. |
| 3.6 | Time horizon(s) | State the time horizon(s) over which costs and consequences are being evaluated | The primary economic analysis will compare the costs and consequences of each arm over the first 26 weeks after randomisation. A secondary analysis will extend this to compare costs and benefits over the two -year follow-up period from randomisation. |
| **Section 4: Economic data collection & management** | | | |
| 4.1 | Statistical software | Specify the statistical software that will be used to carry out the health economic analysis | Stata version x.x or higher will be used for exploratory analysis and Stata and/or MLWin for the main statistical analysis involving multilevel multivariable regression. |
| 4.2 | Identification of resources | Justify and describe items of resource use that will be measured as part of the trial | The following items of health care resource use that may differ between arms will be measured: health service resource use, productivity losses, personal expenditure on health care. In addition staff training and delivery of the intervention will be measured to assess the cost of the intervention. |
| 4.3 | Measurement of resource-use data | Describe the resource-use data collection method(s) (including external routine datasets) and the time points at which they will be used. | Resource-use data will be collected until 18 months post randomisation using trial case report forms (CRFs), completed by the trial nurses. Patient questionnaires (either postal or online) at 6 and 12 months follow up will be used to collect information on other hospital use and community care use. |
| 4.4 | Valuation of resource-use data | For each resource item measured, describe how the unit cost will be derived and from which specific price year. Outline how adjustments will be made for sources from different price years and which inflation index will be used. | All resource use will be valued in monetary terms using appropriate UK unit costs or participant valuations estimated at the time of analysis (2019-2020). Adjustments will be made for inflation using the ONS GDP deflator index. NHS reference costs will be employed to value hospital resource use (e.g. A&E visits and outpatient attendances). Medication costs will be taken from the British National Formulary (BNF) and the Prescription Cost Analysis (PCA) for England. |
| 4.5 | Identification of outcome(s) | Specify and justify the outcome(s) that will be measured | The primary economic outcome measure will be Quality-Adjusted Life Years (QALYs) derived from utility scores, obtained using the EQ-5D-5L quality of life instrument. |
| 4.6 | Measurement of outcome(s) | Describe the outcome data collection method(s) and the time points at which they will be used | Measurements will be recorded prior to randomisation at baseline, and at 2 weeks, 6 weeks and 12 weeks post randomisation. Baseline and research follow-up assessments will take place at the participant's home, general practice or at university premises. |
| 4.7 | Valuation of outcome(s) | For each outcome measured, describe how it will be valued and the source of these valuations | Utility scores will be derived from responses to the EQ-5D-5L. UK utility values will be derived using the approach recommended by NICE, which is currently using the validated mapping function from the existing EQ-5D (-3L). These will be used to form QALYs over the 12-week period, adjusting for any imbalances in baseline EQ-5D-5L scores. |
| **Section 5: Economic data analysis** | | | |
| 5.1 | Analysis population | Outline the analysis population that will be used in the economic base-case analysis (such as intention to treat, per protocol) | The full analysis set will include all randomised participants, which is in accordance with the “intention to treat” (ITT) principle. A per protocol set will include all participants in the full analysis set who are deemed to have no major protocol violations (e.g. patient not receiving any of the intended intervention). |
| 5.2 | Timing of analyses | Describe the timing of all planned analyses (e.g. interim and final analyses) | The primary (“final”) analysis will be conducted once all patients have been followed for two years after the first dose of [trial drug], although an interim analysis will be conducted on year 1 data once all patients have been followed for one year. The interim analysis will take a one-year time horizon and use only data collected in patients’ first year of follow-up, with no extrapolation. The final analysis will include a within-trial analysis, taking a two-year time horizon and extrapolating beyond the end of the trial. |
| 5.3 | Discount rates for costs and benefits | Detail the source of, and justification for, discount rates used for costs and benefits | Costs and benefits will be discounted at 3.5% p.a. as recommended by NICE. |
| 5.4 | Cost-effectiveness threshold(s) | Detail the cost-effectiveness threshold(s) to be used in analysis/interpretation | The estimated mean QALYs and costs associated with each treatment option will be combined with a feasible range of values for decision makers’ willingness-to-pay (ʎ), to obtain the distribution of net benefits at different levels of ʎ. The primary economic analysis will use a cost-effectiveness threshold of £20,000 per QALY. |
| 5.5 | Statistical decision rule(s) | Describe how inference will be drawn (e.g. significance level, confidence intervals or mean net benefit) | Mean differences in costs, QALYs and net benefits between the treatment groups will be estimated with associated 95% confidence intervals. |
| 5.6 | Analysis of resource use | Describe how differences in the use of resources/services between randomised groups will be compared | Differences in the use of services between randomised groups will be described but not compared statistically. |
| 5.7 | Analysis of costs | Describe analyses of the cost data, specifying any covariates for statistical adjustment, assumptions, and alternative methods | Differences in overall mean costs between the arms will be analysed initially using Ordinary Least Squares (OLS) regression adjusting for the minimisation variables of the randomisation procedure. The distribution of residuals from the regression model will then be examined and a decision will be made as to whether OLS is appropriate or another type of regression model should be considered (e.g. Generalised Linear Models (GLM)). |
| 5.8 | Analysis of outcomes | For each outcome used in the economic analysis, describe how the outcome will be analysed, specifying any covariates for statistical adjustment, assumptions, and alternative methods | An appropriate regression model will be used to adjust for any imbalance in baseline utility and the minimisation variables of the randomisation process. |
| 5.9 | Data cleaning for analysis | Outline how data will be cleaned before analysis | Face validity tests will be conducted on data (e.g. to identify misspelt text) and checked against the source documents. Corrections made will be documented in the Stata code. |
| 5.10 | Missing data | Specify the procedure for dealing with missing data | Trial data will be examined for any missing data. The appropriate method for dealing with missing data will depend on the proportion of missing data and likely mechanism of missingness. For example, multiple imputation methods may be used if the data is missing at random (MAR). |
| 5.11 | Analysis of cost-effectiveness | Describe the methods that will be used to summarise cost-effectiveness. | Cost and QALY data will be combined to calculate an incremental cost-effectiveness ratio (ICER) and net monetary benefit (NMB) statistic from the NHS and PSS perspective. Seemingly Unrelated Regression (SUR) will be used, if appropriate, to account for the correlation between the costs and the QALYS. |
| 5.12 | Sampling uncertainty | Describe how uncertainty around the costs and effectiveness estimates and summary cost-effectiveness measures will be explored | The nonparametric bootstrapping approach will be used to determine the level of sampling uncertainty surrounding the mean ICER by generating 10,000 estimates of incremental costs and benefits. |
| 5.13 | Subgroup analyses or analysis of heterogeneity | Describe any analyses of subgroups or heterogeneity in cost-effectiveness and the analysis methods used | Analyses will also be conducted on the final dataset to investigate how cost-effectiveness varies between different patient subgroups (e.g. age <60/60+; sex). Any subgroup analyses for which the smaller subgroup includes fewer than 50 participants will be omitted. |
| 5.14 | Sensitivity analyses | Describe any sensitivity analyses and their form | Several sensitivity analyses will be undertaken to explore uncertainties surrounding key parameters in the economic evaluation. The results for complete cost and quality of life data (i.e. those with no missing data) as well as a strict per-protocol analysis of the data will be provided to identify the impact of missing data on the analysis and any sensitivity to protocol violations. |
| **Section 6: Modelling** | | | |
| 6.1 | Extrapolation or decision analytic modelling | Outline whether decision analytic modelling or any other extrapolation will be used to estimate cost-effectiveness results beyond the period of the trial or to introduce an additional comparator or other evidence. | Decision analytic modelling will be undertaken (e.g. to extrapolate costs & outcomes beyond the follow up period of trial), irrespective of statistical significance in trial results, if there is potential for the cost -effectiveness of active interventions to improve under a longer analysis time horizon than the 18 months follow-up and /or under a more efficient risk screening process. |
| 6.2 | Model type | Describe the modelling approach that will be used and duration of extrapolation | A long-term Markov decision model with annual cycle will be used to evaluate effects of intervention on costs, health gains and cost-effectiveness over the patient’s lifetime. |
| 6.3 | Model structure | Detail the model structure (where possible, include diagram of model states and transitions between them) | The decision model will comprise three arms, with one arm for each intervention evaluated. Each arm will be structured as a Markov model built around health states to which health care cost and health related quality of life (HRQoL) data collected as part of the trial will be linked. The model structure is shown in Figure X. |
| 6.4 | Treatment effect beyond the end of the trial | Describe the duration and size of treatment effect in the period beyond the end of the trial | The hazard ratio for disease specific mortality observed during the trial will be assumed to persist after the end of trial follow up. |
| 6.5 | Other key assumptions | List the key structural assumptions of the model | The main structural assumptions underpinning the decision model are listed below [list those that are relevant to specific disease/trial]. The model structure maybe subject to change, following initial exploratory analysis of trial data. |
| 6.6 | Methods for identifying and estimating parameters | For each model parameter, describe the methods and data sources that will be used to estimate the parameter (e.g. from the RCT, systematic review, meta-analysis, other published data or expert opinion) | The model will require 4 main sets of parameters, which are expected to be mainly derived from the trial data. 1) Transition probabilities between health states, 2) Treatment Effect/s of the intervention, 3) Quality of life decrements, 4) Health care costs. Transition probabilities between health states will be estimated by sex and 5 or 10-year age groups using trial data. Quality of life decrements and costs associated with a fall or fracture will be derived from patient questionnaires, analysed using regression analysis and routine hospital admissions data.  Other parameters, for example related to ‘natural history of disease’, will be parameterised using published data, and where necessary using formally elicited expert opinion. |
| 6.7 | Model uncertainty | Describe the methods that will be used to assess parameter uncertainty in the results. Describe sensitivity analyses for the impact of other types of uncertainty on results. | Parameter uncertainty will be assessed using probabilistic sensitivity analysis (i.e. by fitting a probability distribution to each uncertain parameter) and running Monte Carlo simulations |
| 6.8 | Model validation | Describe the methods and data that will be used to check the face, internal and external validity of the model | The model will be estimated and internally validated using trial data. |
| 6.9 | Subgroup analyses/heterogeneity | Describe subgroup or heterogeneity analyses that will be executed and reported within the extrapolation or decision analytic modelling | The model will be used to evaluate the cost-effectiveness of the intervention in categories of trial participants by age (50-59; 60-69; 70+) and estimated 5-year vascular disease risk (<10%; ≥10,<20%; ≥20%). |
| **Section 7: Reporting/publishing** | | | |
| 7.1 | Reporting standards | Describe any guidelines that will be followed when publishing results | CHEERS guidelines will be followed when reporting the health economic evaluation, in a format appropriate to stakeholders and policy makers. |
| 7.2 | Deviations from the HEAP | Describe the procedure for reporting any deviations from the HEAP | Any deviation from HEAP will be described and justified in the final published report. |
| **Section 8: Appendices** | | | |
| 8.1 | Health economic collection tools | Include template examples of the resource-use data collection sheets and resource-use questionnaires | Data collection sheets on hospital stays, visits to A&E, and home visits are given in Appendix X and include health utility measures. The resource-use questionnaire has been deposited in DIRUM (http://www.dirum.org/). |

**Optional items**

|  |  | **Description** | **Example** |
| --- | --- | --- | --- |
| **Section 1: Administrative information** | | | |
| O1.1 | Table of contents | List of HEAP contents with page numbers |  |
| O1.2 | Abbreviations/glossary of terms/definitions | List of abbreviations and/or acronyms used within the HEAP alongside their meanings/definitions | QALY: quality-adjusted life year  NHS: National Health Service |
| **Section 4: Economic data collection & management** | | | |
| O4.1 | Monitoring collection of health economic data | Outline how the health economic data collected will be monitored | Training will be provided to individuals responsible for administering the health economics questionnaires. The trial health economist(s) will work closely with the trial team throughout the data collection period. Data collection forms will be assessed throughout the trial period to monitor quality of the data and amend any forms or procedures if necessary. |
| O4.2 | Database management | Outline how the economic data will be stored and managed and by whom | Economic data will be securely stored on the trial database and managed by the trial database manager, [name(s)]. |
| O4.3 | Data entry | Outline how data will be entered/handled and outline any checking systems in place | All baseline data will be entered into the case report form (CRF) by the trial research nurses at the recruitment site. Follow-up data collected from postal questionnaires will be entered by the central research team. The database will use controls to limit data entry to plausible values. |
| O4.4 | Data archiving | State whether datasets, interim datasets and final analysis will be archived, and if so, how | A copy of health economic analysis files, derived datasets, interim datasets and final analysis will be locked and archived. Archived datasets will be held by [organisation name] and will conform to the department data security policy and department data compliance and Data Protection Act policies. |
| **Section 6: Modelling** | | | |
| O6.1 | Value of information analysis | Describe whether value of information analysis is planned and the type and methods that will be used to calculate value of information | As part of the analysis we will conduct a value of information analysis (VOI). Expected value of perfect information (EVPI) will be used to indicate whether the research is potentially worthwhile. In addition, the expected value of perfect information for parameters (EVPPI) will also be calculated to identify those parameters for which more precise estimates would be most valuable. |
| **Section 8: Appendices** | | | |
| O8.1 | Cross-referencing to other trial documents | Reference to other relevant trial documents that are adhered to and followed when writing the HEAP and any other references used when writing the HEAP | The [name of trials unit] Standard Operating Procedure for Economic Evaluations (version 2.1) was followed in designing this analysis. The analysis described in this plan adheres to the [organisation name] Data Management Plan (version 1.1). |
| O8.2 | Illustrations | Illustrations such as annotated questionnaires detailing the database fieldnames, flow charts outlining the flow of data for the economic evaluation, or template tables | The conduct and procedures for the economic evaluation will be fully integrated into the [name] trial from planning and designing the economic evaluation through to publication. The following flowchart should be applied. |

**†** Examples were extracted, and in some cases modified, from existing draft or final HEAPs provided to the study team. We are grateful to the trial teams from which they originated.
